# Cardiac SGLT2 Expression and Cell-Type-Specific Responses to Empagliflozin in iPSC-Derived Models of Diabetic Cardiomyopathy

**DOI:** 10.3390/jcdd13070341

**Published:** 2026-07-21

**Authors:** Nan Su, Ren Jie Phang, Anne M. Kong, Richard J. MacIsaac, Shiang Y. Lim, Jarmon G. Lees

**Affiliations:** 1 O’Brien Institute Department, St Vincent’s Institute of Medical Research, Fitzroy, VIC 3065, Australiamlim@svi.edu.au (S.Y.L.); 2 Department of Medicine at St Vincent’s Hospital, Melbourne Medical School, The University of Melbourne, Melbourne, VIC 3010, Australia; richard.macisaac@svha.org.au; 3 Department of Endocrinology & Diabetes, St Vincent’s Hospital Melbourne, Fitzroy, VIC 3065, Australia; 4 Australian Centre for Accelerating Diabetes Innovations, School of Medicine, The University of Melbourne, Melbourne, VIC 3010, Australia; 5 Department of Surgery, Melbourne Medical School, The University of Melbourne, Melbourne, VIC 3010, Australia; 6 Drug Discovery Biology, Monash Institute of Pharmaceutical Sciences, Monash University, Melbourne, VIC 3010, Australia; 7 National Heart Research Institute Singapore, National Heart Centre, Singapore 169609, Singapore

**Keywords:** SGLT2 inhibitor, empagliflozin, diabetic cardiomyopathy, type 2 diabetes, cardiac microtissue

## Abstract

Sodium–glucose co-transporter 2 (SGLT2) inhibitors confer cardioprotection in patients with and without diabetes; however, whether SGLT2 is expressed in cardiac tissue and how these drugs act locally in the heart remains unclear. We investigated SGLT2 expression and the effects of empagliflozin in human iPSC-derived cardiac cells exposed to diabetogenic conditions. SGLT2 expression and the effects of empagliflozin were assessed in iPSC-derived cardiomyocytes, endothelial cells, and cardiac fibroblasts under acute diabetogenic conditions using protein expression and metabolic activity assays, and in a multicellular 3D cardiac microtissue model using metabolic activity and contraction analyses. SGLT2 was detected in all three iPSC-derived cardiac cell types with nuclear and perinuclear localisation; no membrane-bound expression was observed. Endothelial cell SGLT2 expression was elevated under diabetogenic conditions. Diabetogenic stress reduced metabolic activity in both cardiomyocytes and endothelial cells; empagliflozin partially rescued endothelial cell metabolic activity but had no effect in cardiomyocytes. Empagliflozin reversed diabetogenic stress-induced cardiac fibroblast activation. 3D cardiac microtissues under diabetogenic conditions exhibited prolonged relaxation time, reduced beat rate variability, and reduced metabolic activity. Empagliflozin maintained metabolic activity at levels comparable to those of the control but did not rescue relaxation time or beat rate variability. The responsiveness of non-myocytes (endothelial cells and cardiac fibroblasts) to empagliflozin, in the absence of any effect on cardiomyocytes, suggests that non-myocyte-mediated mechanisms may contribute to the clinically observed cardioprotection of SGLT2 inhibitors.

## 1. Introduction

Diabetes is characterised by chronic hyperglycaemia resulting from blood glucose dysregulation and pancreatic dysfunction [1]. Individuals with Type 2 Diabetes (T2D) are at a higher risk of developing a specific form of heart disease known as diabetic cardiomyopathy [2]. Diabetic cardiomyopathy is a diabetes-induced heart disease that occurs in the absence of pre-existing cardiac conditions such as hypertension or coronary artery disease [3]. As one of the most severe complications of T2D, it affects over 30% of T2D individuals [4]. The condition carries a grave prognosis, exceeding that of metastatic breast cancer, with a 12.5% mortality rate within five years of onset [5].

Sodium–glucose co-transporter 2 (SGLT2) inhibitors, such as empagliflozin, are a common treatment option for glycaemic control in individuals with diabetes [6]. These drugs have been shown to not only improve glycaemic control but also reduce patient hospitalisation and mortality from cardiovascular events in both diabetic and non-diabetic patients [7,8]. However, the mechanism by which SGLT2 inhibitors confer cardioprotection remains unclear. There is also a lack of consensus on whether SGLT2 is expressed in the heart.

SGLT2 is a well-characterised membrane transporter responsible for ~90% of total daily glucose reabsorption in the renal proximal tubule [9]. Until recently, it was believed that SGLT2 was primarily expressed in renal tissue, with some potential expression in pancreatic α-cells, but not in the human heart [10,11,12,13]. The direct cardioprotective effect of SGLT2 inhibitors via SGLT2 protein has generated considerable debate. Empagliflozin has been postulated to exert cardioprotective effects through mechanisms including cardiac sodium–hydrogen exchanger-1 inhibition, autophagy, SGLT1 inhibition and inflammatory regulation [14,15,16,17,18]. However, none of these studies directly relate to cardiac SGLT2 expression. A recent study found SGLT2 expression in the heart tissue of individuals with late-stage cardiomyopathy, in both diabetic and non-diabetic individuals [19]. Healthy hearts transplanted into diabetic individuals showed a significant increase in SGLT2 expression, and hyperglycaemia induced SGLT2 expression in primary human cardiomyocytes [19]. These findings call for a re-evaluation of the cardioprotective effects of SGLT2 inhibitors and underscore the need for further investigation into their mechanisms of action to optimise drug development. Notably, whether these effects are mediated through cardiomyocytes alone or also through non-myocyte populations, including endothelial cells and cardiac fibroblasts, remains poorly characterised.

Human-induced pluripotent stem cells (iPSCs) can self-renew indefinitely and differentiate into most specialised cell types in the human body, enabling studies to be conducted in a human-specific context [20]. Furthermore, human iPSCs can be bioengineered into 3D tissue models, which better represent human tissues and organs compared to traditional 2D monolayer cultures [21].

In this study, we detected SGLT2 expression in human iPSC-derived cardiomyocytes, endothelial cells, and cardiac fibroblasts. We also showed direct responsiveness of endothelial cells and cardiac fibroblasts towards SGLT2 inhibitors under acute diabetogenic conditions. Lastly, our 3D-engineered cardiac microtissue exhibited an early-stage diabetic cardiomyopathy phenotype through altered contraction profiles under diabetogenic stress. Together, these findings implicate non-cardiomyocyte populations as potential mediators of SGLT2 inhibitor cardioprotection and establish human iPSC-derived cardiac models as a platform for investigating the direct cardiac actions of these drugs.

## 2. Materials and Methods

### 2.1. Human Induced Pluripotent Stem Cell (iPSC) Culture

The human iPSC-Foreskin-2 (CL2) cell line, kindly provided by James A. Thomson (University of Wisconsin) [22], was maintained on vitronectin-coated plates (Thermo Fisher Scientific, Waltham, MA, USA) in TeSR-E8 medium (Stem Cell Technologies, Vancouver, BC, Canada) according to the manufacturer’s protocol. The culture medium was refreshed every two to three days, and the cells were passaged on a weekly basis using ReLeSR (Stem Cell Technologies).

### 2.2. Cardiomyocyte Differentiation

Cardiomyocytes were differentiated from CL2 human iPSCs as previously described [23]. Briefly, cardiomyocyte specification was induced through sequential Wnt pathway activation and inhibition, followed by metabolic purification in glucose-free, lactate-supplemented medium from day 14 to day 19. On day 19, 25,000 cardiomyocytes/cm^2^ were passaged into DMEM/F-12 GlutaMAX medium supplemented with 20% foetal calf serum (Sigma-Aldrich, St. Louis, MO, USA), 0.1 mM 2-mercaptoethanol, 0.1 mM nonessential amino acids, 50 U/mL penicillin/streptomycin, and 10 µM Y-27632 dihydrochloride. Cardiomyocyte purity was confirmed by cardiac troponin T (cTnT) staining; only batches exceeding 90% cTnT-positive cells were used. On day 20, the medium was changed to RPMI/B27 (no glucose) supplemented with 20 ng/mL L-ascorbic acid and 5.55 mM glucose for 24 h. On day 21, iPSC-derived cardiomyocytes were subjected concurrently for 48 h to either control conditions (5.55 mM glucose and 14.45 mM mannitol in RPMI/B27 (no glucose) + 20 ng/mL L-ascorbic acid + 1× B27 supplement), acute diabetogenic conditions (20 mM glucose, 0.25 mM palmitate, 0.1 mM linoleic acid, 0.1 mM oleic acid, 10 nM endothelin-1 and 1 µM cortisol in RPMI/B27 (no glucose) + L-ascorbic acid + 1× B27), or diabetogenic conditions with empagliflozin (Selleckchem, Houston, TX, USA) at 0.1, 1, or 10 µM. The diabetogenic medium was adapted from diabetogenic medium established in previous studies [23,24,25] and adapted to include physiologically relevant levels of fatty acids [26,27]. The empagliflozin dose range was selected to cover clinically relevant concentrations based on published pharmacokinetic data [28,29].

### 2.3. Endothelial Cell Differentiation

CD31+ endothelial cells were differentiated from CL2 iPSCs as previously described [30,31]. Sorted CD31+ cells were expanded in EGM-2MV medium on fibronectin-coated flasks and used at passage 1 (P1). At P1, endothelial cells were seeded at 25,000 cells/cm^2^ on 10 µg/mL human fibronectin and subjected concurrently for 48 h to either control conditions (5.55 mM glucose and 14.45 mM mannitol in EGM-2MV), acute diabetogenic conditions (20 mM glucose, 0.25 mM palmitate, 0.1 mM linoleic acid, 0.1 mM oleic acid, 10 nM endothelin-1 and 1 µM cortisol in EGM-2MV), or diabetogenic conditions with empagliflozin at 0.1, 1, or 10 µM.

### 2.4. Cardiac Fibroblast Differentiation

Cardiac fibroblasts were differentiated from CL2 iPSCs as previously described [23]. On day 29, cardiac fibroblasts were seeded at 25,000 cells/cm^2^ on 0.1% porcine gelatine and subjected concurrently for 48 h to either control conditions (10.01 mM glucose and 9.99 mM mannitol in FGM3), acute diabetogenic conditions (20 mM glucose, 0.25 mM palmitate, 0.1 mM linoleic acid, 0.1 mM oleic acid, 10 nM endothelin-1 and 1 µM cortisol in FGM3), or diabetogenic conditions with 10 µM empagliflozin. Note that the proprietary FGM3 medium contains 10.01 mM glucose.

### 2.5. Cardiac Microtissue Construction

Cardiac microtissues were constructed in accordance with protocols described previously [25,32,33,34]. A total of 140,000 purified day-19 iPSC-derived cardiomyocytes were plated onto 0.1% gelatine-coated (Sigma-Aldrich) 48-well Nunc™ UpCell plates (Thermo Fisher Scientific) in DMEM/F-12 GlutaMAX medium supplemented with 20% foetal calf serum (Sigma-Aldrich), 0.1 mM 2-mercaptoethanol, 0.1 mM nonessential amino acids, 50 U/mL penicillin/streptomycin, and 10 µM Y-27632 dihydrochloride. After 24 h, 55,000 iPSC-derived endothelial cells and 5000 iPSC-derived cardiac fibroblasts were seeded on top of the cardiomyocytes and cultured in cardiac microtissue medium supplemented with Y-27632. Cardiac microtissue medium consisted of a 1:1:1 mixture of RPMI/B27 (no glucose), EGM2-MV, and FGM3, with the final glucose concentration adjusted to 5.55 mM and supplemented with 50 ng/mL VEGF-165. After 24 h, the 48-well Nunc™ UpCell plates were brought to room temperature to detach the cell sheet, which was then transferred to an ultralow attachment plate (Sigma-Aldrich) and maintained in cardiac microtissue medium supplemented with Y-27632 for 24 h. Subsequently, the cardiac microtissues were embedded in 10 µL of growth factor-reduced Matrigel and cultured in cardiac microtissue medium in a humidified CO_2_ incubator on an orbital shaker at 60 rpm, with the medium refreshed every 2–3 days. Four days after the initial cardiac microtissue construction, they were subjected to either control conditions (5.55 mM glucose and 14.45 mM mannitol), diabetogenic conditions (20 mM glucose, 0.25 mM palmitate, 0.1 mM linoleic acid, 0.1 mM oleic acid, 10 nM endothelin-1 and 1 µM cortisol), or diabetogenic conditions for 48 h. Subsequently, 10 µM empagliflozin was administered to the diabetogenic group for an additional 48 h as a rescue model. All cardiac microtissues received a total treatment duration of 96 h.

### 2.6. Cell Death

Cell death was assessed by staining the cells with propidium iodide (5 µg/mL) and Hoechst 33258 (1 µg/mL; Sigma-Aldrich) nucleic acid stain in Hank’s Buffer Saline Solution with Mg^2+^ and Ca^2+^ (HBSS+/+; Sigma-Aldrich). The cells were incubated for 45 min at 37 °C in the dark, followed by two washes with HBSS+/+ and imaging in HBSS+/+ solution. Fluorescent images were captured from five randomly selected fields using a fluorescence microscope (Olympus IX71, Olympus Corporation, Tokyo, Japan) at 20× magnification. The number of cells positive for Hoechst 33258 and propidium iodide was quantified using the Cell Counter plugin in ImageJ software (version 1.54t). A minimum of 1000 total cells were counted per data point.

### 2.7. Cell Metabolic Activity

The metabolic activity of cells was assessed using the CellTiter-Blue Cell Viability Assay (Promega, Madison, WI, USA). Cardiomyocytes and endothelial cells were incubated with CellTiter-Blue reagent and cell-specific culture medium in a 1:5 mixture in a 96-Well White Polystyrene Microplate (Corning, Corning, NY, USA) at 37 °C in the dark for 4 h, after which the fluorescence was immediately recorded at 560/590 nm using an EnSpire^®^ Multimode Plate Reader (PerkinElmer, Shelton, CT, USA).

### 2.8. Immunocytochemistry

Cells were fixed on glass coverslips with 10% neutral buffered formalin (Amber Scientific, Alvarado, TX, USA) for 15 min at room temperature, permeabilised with 0.2% Triton X-100 (Sigma-Aldrich) for 10 min, blocked with protein block (Thermo Fisher Scientific) for 10 min, and subsequently labelled overnight at 4 °C with cardiac troponin T (10 µg/mL, mouse monoclonal, ab8295, Abcam, Waltham, MA, USA), CD31 (20 µg/mL, mouse monoclonal, M0823, Dako, Glostrup, Denmark), SGLT2 (2 µg/mL, rabbit monoclonal, ab37296, Abcam) or vimentin (0.3 µg/mL, mouse monoclonal, M072529-2, Dako), followed by Alexa Fluor-594-conjugated goat anti-rabbit IgG (2 µg/mL, A-11012, Invitrogen, Carlsbad, CA, USA) or Alexa Fluor-488-conjugated goat anti-mouse IgG (2 µg/mL, A-11001, Invitrogen) for 1 h at room temperature. All antibodies were diluted in EnVision Flex Antibody Diluent (Dako). DAPI (1 µg/mL; Sigma-Aldrich) was added together with the secondary antibodies for nuclear staining. Glass coverslips were mounted with a fluorescence mounting agent (Dako). Images were captured using a fluorescence microscope (Olympus BX61, Olympus Corporation, Tokyo, Japan).

### 2.9. Cardiac Fibroblast Activation

Cardiac fibroblast activation was assessed according to a previously published protocol [35]. Following treatment, cardiac fibroblasts were fixed, permeabilised, and blocked according to the protocol described above, then stained for αSMA (3.33 µg/mL, mouse monoclonal, 1A4, Abcam) and DAPI using an Alexa Fluor-488-conjugated goat anti-mouse IgG secondary antibody (2 µg/mL, A-11001, Invitrogen). Fluorescent images were captured from five random fields using a fluorescence microscope (Olympus IX71) at 20× magnification, and the percentage of αSMA-positive cells were quantified relative to the total number of DAPI-positive nuclei using the Cell Counter plugin in ImageJ.

### 2.10. Western Blotting

Treated cells were lysed in RIPA buffer (Abcam) with protease inhibitor and clarified at 12,000× *g* for 15 min at 4 °C. Total protein concentration was determined using the Pierce BCA protein assay kit (Thermo Fisher Scientific). A total of 60 µg protein per sample was electrophoresed on an NuPAGE 4–12% Bis-Tris Mini Protein Gel (Thermo Fisher Scientific) at 150 V for 60 min and transferred for 90 min at 30 V. Membranes were blocked with Odyssey Blocking Buffer (LI-COR Biosciences, Lincoln, NE, USA) for 1 h at room temperature, then co-incubated with SGLT2 (1:100; rabbit; 24654-1-AP; Proteintech, Rosemont, IL, USA) and calnexin (1:2000; rabbit; C4731; Sigma-Aldrich) primary antibodies for 24 h at 4 °C. Following incubation with AlexaFluor 680 donkey anti-rabbit secondary antibody (1:20,000; A10043; Thermo Fisher Scientific), membranes were visualised on an Odyssey CLx Imager (LI-COR Biosciences). SGLT2 and calnexin bands were resolved at ~46–75 kDa and ~90 kDa, respectively. Densitometry of target protein bands were performed using Image Studio Software (version 6.0.0.28); SGLT2 expression was normalised to calnexin for quantification.

### 2.11. Cardiac Microtissue Contraction Analysis

Videos for contraction analysis were taken using a bright field microscope (Olympus IX71) at 60 frames per second for 15 s. The contraction videos were imported into ImageJ software, and contraction was assessed using the MUSCLEMOTION plugin [36] to assess contraction duration, time-to-peak, and relaxation time. Variability of the beat rate was calculated as the root mean square of successive differences (RMSSDs) of the peak-to-peak intervals [37].

### 2.12. Cardiac Microtissue Metabolic Activity

Metabolic activity of the microtissues was assessed using the CellTiter-Glo Luminescent Cell Viability Assay (Promega). Microtissues were equilibrated to room temperature in a 96-well plate for 30 min before the medium was replaced with CellTiter-Glo 3D reagent dye mixed with conditioned medium at a 1:1 ratio. The solution was then homogenised using the mixing function on a plate reader for 5 min at high speed. Microtissues were incubated at room temperature for 25 min to stabilise the luminescence signal before it was recorded using a POLARStar (BMG Labtech, Ortenberg, Germany).

### 2.13. Statistics

Data are presented as the mean ± standard error of the mean (SEM). Statistical analyses were performed using GraphPad Prism (version 10.4.2633) using either a Student’s *t*-test or one-way ANOVA followed by Dunnett’s or Tukey’s post hoc test, where appropriate. *p* < 0.05 was considered statistically significant. Only *p*-values < 0.05 are shown on the graphs. Outliers in the cardiac microtissue contraction data were identified and removed using the ROUT method (Q = 10%) in GraphPad Prism. *n* is reported as the number of individual wells/microtissues assessed across independent experiments.

## 3. Results

### 3.1. SGLT2 Expression in Cardiac Cells

**Figure 1:**
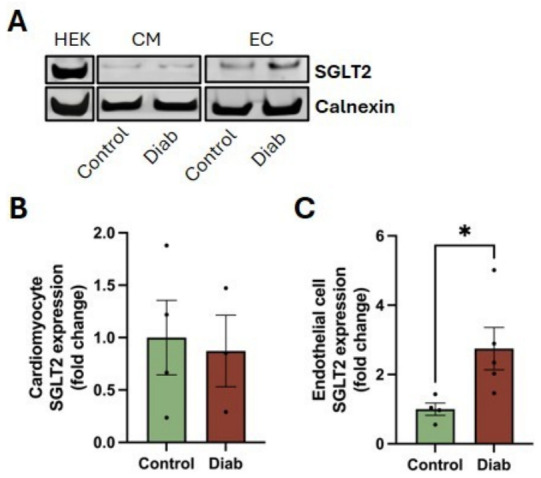
SGLT2 protein expression in human iPSC-derived cardiomyocytes and endothelial cells. (**A**) Representative Western blot showing SGLT2 expression in cardiomyocytes (CM) and endothelial cells (EC) under control and diabetogenic (Diab) conditions. Human embryonic kidney (HEK) cells were included as a positive control. (**B**,**C**) Densitometry of SGLT2 protein expression in cardiomyocytes (**B**) and endothelial cells (**C**) following 48 h under control or diabetogenic conditions, normalised to calnexin. Data are presented as mean ± SEM; *n* = 3–4 independent experiments (**B**) and *n* = 4–5 independent experiments (**C**). * *p* < 0.05 by unpaired *t*-test. Calnexin: ~90 kDa; SGLT2: ~46–75 kDa.

To determine whether SGLT2 protein is expressed in iPSC-derived cardiac cells and whether its expression is modulated by diabetogenic stress, Western blotting was performed in cardiomyocytes and endothelial cells. SGLT2 protein was detected under both control and diabetogenic conditions in both cell types (Figure 1A and Figure S1). Expression was unchanged in cardiomyocytes regardless of condition (Figure 1B), whereas diabetogenic stress increased SGLT2 expression 3.1-fold in endothelial cells compared to the control (Figure 1C).

### 3.2. Intracellular Localisation of SGLT2 in Human iPSC-Derived Cardiomyocytes, Endothelial Cells, and Cardiac Fibroblasts

**Figure 2:**
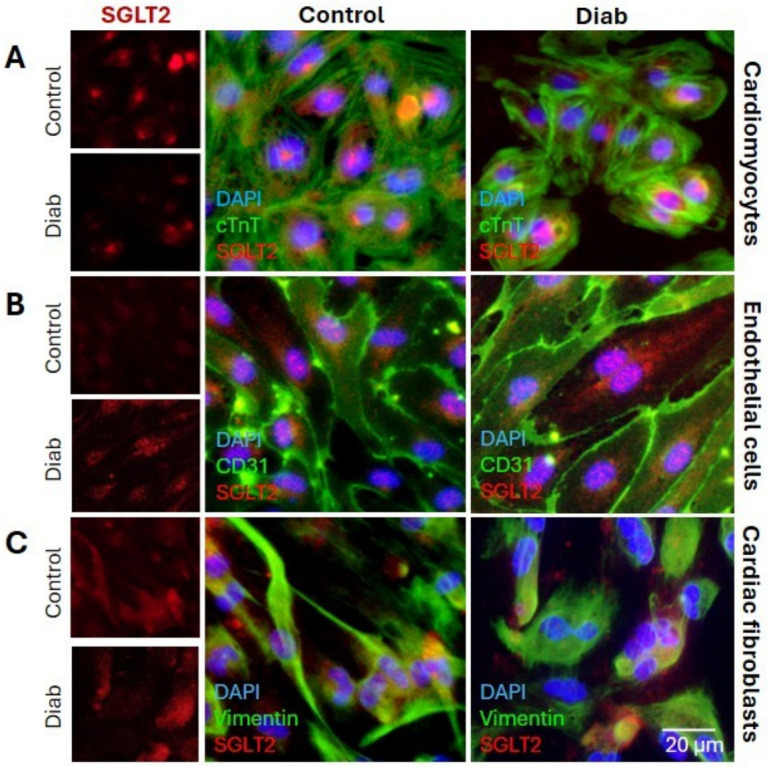
SGLT2 protein localisation in iPSC-derived cardiomyocytes, endothelial cells, and cardiac fibroblasts. (**A**–**C**) Representative immunofluorescence images of SGLT2 (red) localisation in cardiomyocytes co-stained with cTnT (green; (**A**)), endothelial cells co-stained with CD31 (green; (**B**)), and cardiac fibroblasts co-stained with vimentin (green; (**C**)), under control or diabetogenic (Diab) conditions for 48 h. Cell nuclei were counterstained with DAPI (blue). Scale bar = 20 µm.

SGLT2 immunostaining revealed a consistent localisation pattern across all three cell types under both control and diabetogenic conditions (Figure 2A–C): punctate nuclear and perinuclear expression with some diffuse cytoplasmic signal, unchanged by diabetogenic stress. No membrane-bound SGLT2 was detected in any cell type. This intracellular localisation is consistent with that reported in primary human cardiomyocytes [19], supporting the relevance of iPSC-derived cardiac cells for investigating cardiac SGLT2.

### 3.3. SGLT2 Inhibitors Increase Metabolic Activity in Endothelial Cells but Not Cardiomyocytes Under Diabetogenic Conditions

**Figure 3:**
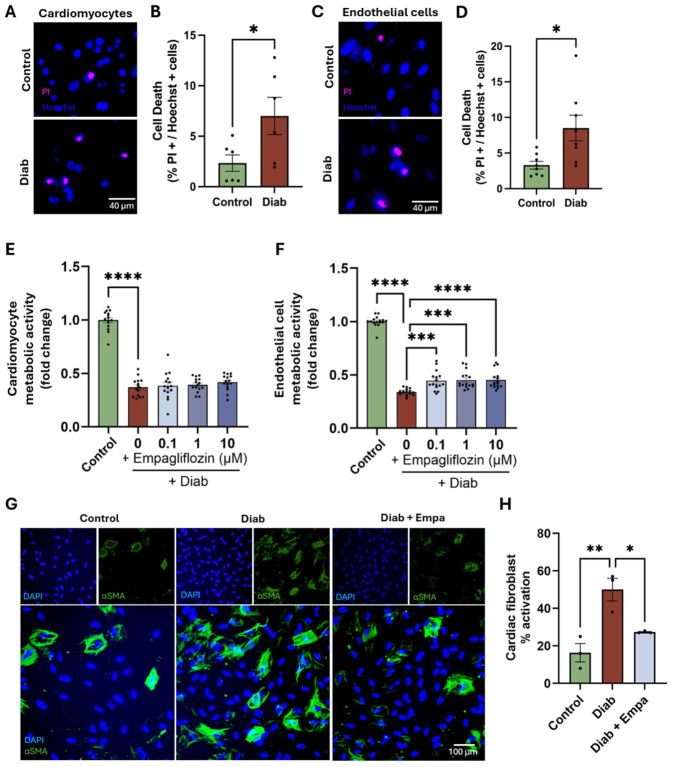
Therapeutic effects of empagliflozin on cardiomyocytes, endothelial cells, and cardiac fibroblasts. (**A**,**C**) Representative images of cardiomyocytes (**A**) and endothelial cells (**C**) under control or diabetogenic (Diab) conditions, stained with propidium iodide (PI; purple) and Hoechst 33258 (blue). Scale bar = 40 µm. (**B**,**D**) Quantification of cell death in cardiomyocytes (**B**) and endothelial cells (**D**), expressed as the percentage of PI-positive/Hoechst-positive cells; *n* = 6–8 from three to four independent experiments. * *p* < 0.05 by unpaired *t*-test. (**E**,**F**) Cell metabolic activity assessed by the CellTiter-Blue in cardiomyocytes (**E**) and endothelial cells (**F**) under control, diabetogenic, or diabetogenic + empagliflozin (Empa; 0.1, 1, or 10 µM) conditions for 48 h; *n* = 15 from six independent experiments. * *p* < 0.05, *** *p* < 0.001, and **** *p* < 0.0001 vs. the diabetogenic group by one-way ANOVA with Dunnett’s post hoc test. (**G**) Representative immunofluorescence images of cardiac fibroblasts stained for αSMA (green) and DAPI (blue) under control, diabetogenic, and diabetogenic + empagliflozin (10 µM) conditions. (**H**) Quantification of cardiac fibroblast activation (percentage of αSMA-positive cells); *n* = 3 independent experiments. * *p* < 0.05 and ** *p* < 0.01 vs. the diabetogenic group by one-way ANOVA with Tukey’s post hoc test. Data are presented as mean ± SEM.

To confirm that diabetogenic conditions induced a measurable pathological response prior to drug treatment assessments, cell death was quantified by propidium iodide and Hoechst co-staining. Diabetogenic conditions significantly increased cell death in both cardiomyocytes (+4.7% ± 2.0%; Figure 3A,B) and endothelial cells (+5.2% ± 1.9%; Figure 3C,D) compared with the control.

Cardiomyocyte metabolic activity was reduced 0.6-fold under diabetogenic conditions; however, empagliflozin had no significant effect at any dose examined (0.1, 1, and 10 µM; Figure 3E), suggesting that cardiomyocytes are not a direct cellular target under these conditions. In contrast, endothelial cell metabolic activity was similarly reduced 0.6-fold under diabetogenic conditions, and concurrent empagliflozin treatment significantly rescued metabolic activity at all doses (10.5–11.2% increase), with the greatest effect observed at 10 µM (Figure 3F). Based on this dose–response relationship, 10 µM empagliflozin was used for subsequent fibroblast and microtissue experiments.

Diabetogenic conditions increased cardiac fibroblast activation by 33.7% ± 16.9% compared with the control (Figure 3G,H), consistent with the profibrotic response observed in diabetic cardiomyopathy. Treatment with 10 µM empagliflozin significantly reduced the proportion of activated fibroblasts to levels comparable to those of the control (Figure 3H).

### 3.4. 3D Cardiac Microtissues Recapitulate Early Diabetic Cardiomyopathy Phenotypes with Limited Empagliflozin Responsiveness

**Figure 4:**
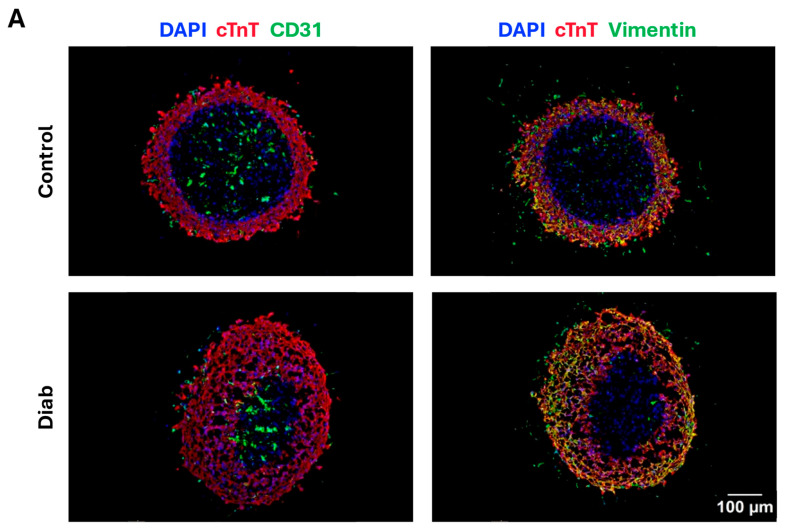

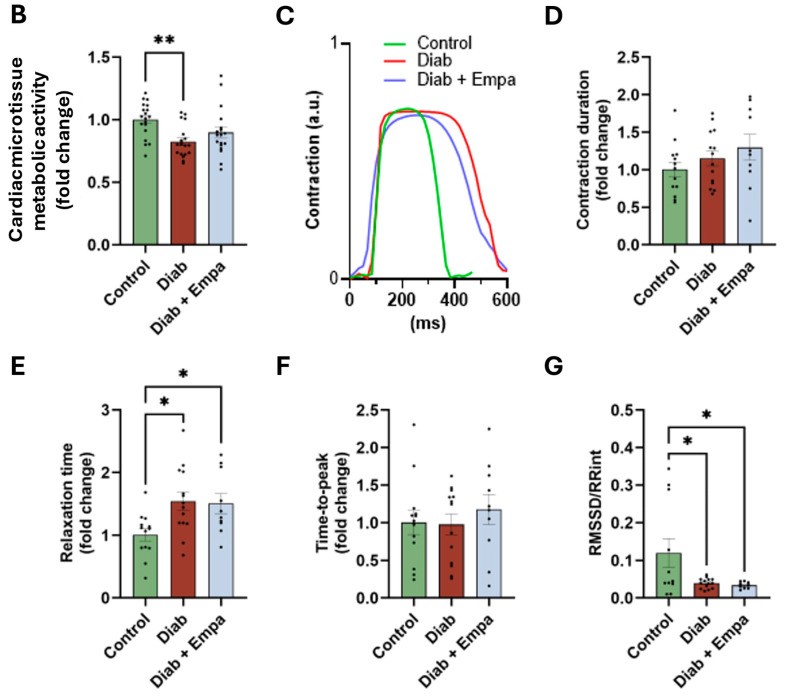
Therapeutic effects of empagliflozin on 3D cardiac microtissues. (**A**) Representative immunofluorescence images of cardiac microtissues under control or diabetogenic (Diab) conditions showing cardiomyocytes (cTnT, red), endothelial cells (CD31, green), and cardiac fibroblasts (vimentin, green). Cell nuclei were counterstained with DAPI (blue). Scale bar = 100 µm. (**B**) Metabolic activity assessed by CellTiter-Glo assay. Microtissues were cultured under control or diabetogenic conditions for 48 h, after which empagliflozin (Empa; 10 µM) or vehicle control was introduced as a rescue intervention for an additional 48 h. (**C**) Representative single-cycle contraction traces per group. (**D**–**G**) Quantification of contraction duration (**D**), relaxation time (**E**), time-to-peak (**F**), and beat rate variability (RMSSD/RRint; G). Data are presented as mean ± SEM; *n* = 10–16 from five independent experiments. * *p* < 0.05 and ** *p* < 0.01 by one-way ANOVA with Tukey’s post hoc test.

To extend our 2D findings, we employed an established 3D multicellular cardiac microtissue model comprising cardiomyocytes, endothelial cells, and cardiac fibroblasts [23,25] using a rescue design. Microtissues were exposed to diabetogenic conditions for 48 h, after which empagliflozin (10 µM) was introduced for a further 48 h to assess its capacity to reverse an established diabetogenic phenotype. All tissues were assessed at 96 h. Immunofluorescence imaging revealed cTnT-positive cardiomyocytes organised at the microtissue periphery, with CD31-positive endothelial cells distributed throughout the tissue interior (Figure 4A). Vimentin-positive cardiac fibroblasts were predominantly localised in close association with cardiomyocytes in an interstitial distribution. Microtissue cytoarchitecture was broadly similar between the control and diabetogenic conditions.

At the 48 h baseline, prior to empagliflozin treatment, contraction duration, relaxation time, time-to-peak, and beat rate variability did not differ significantly between control and diabetogenic microtissues (Supplementary Figure S2A–D).

Diabetogenic conditions significantly reduced microtissue metabolic activity at 96 h compared with the control (Figure 4B). Empagliflozin-treated microtissues maintained metabolic activity at levels comparable to control, in contrast to the significant reduction observed under diabetogenic conditions alone.

Representative contraction traces at 96 h revealed a visibly prolonged contraction profile in diabetogenic microtissues, driven primarily by elongation of the relaxation phase (Figure 4C; Supplementary Videos S1 and S2). Quantitative analysis confirmed significantly prolonged relaxation time under diabetogenic conditions (Figure 4E), while total contraction duration (Figure 4D) and time-to-peak (Figure 4F) were unaffected. Beat rate variability (RMSSD/RRint) was significantly reduced under diabetogenic conditions compared with the control (Figure 4G), consistent with reduced heart rate variability observed clinically in diabetic cardiomyopathy. Empagliflozin did not rescue relaxation time, beat rate variability, or any other contractile parameter assessed (Figure 4D–G).

## 4. Discussion

SGLT2 inhibitors have shown cardioprotection in individuals regardless of their diabetic status [38,39,40], yet their mechanism of action remains unclear. We employed human iPSC-derived cardiomyocytes, endothelial cells and cardiac fibroblasts to model diabetic cardiomyopathy in a human context and examined the cardiac expression of SGLT2 and its potential cardioprotective mechanism of action. SGLT2 expression was observed in human cardiovascular cells, including cardiomyocytes, endothelial cells, and cardiac fibroblasts. Empagliflozin significantly increased endothelial cell metabolic activity under diabetogenic conditions, but cardiomyocytes subjected to diabetogenic stress did not exhibit any signs of protection. In contrast, diabetogenic-induced cardiac fibroblast activation was reversed using empagliflozin. Finally, using the 3D multicellular cardiac microtissue model of diabetic cardiomyopathy established in our previous work [23,25], we observed early-stage contractile dysfunction and reduced metabolic activity under diabetogenic conditions. Empagliflozin maintained metabolic activity at levels comparable to those of the control but did not rescue contractile dysfunction at the single dose examined, contrasting with the broader efficacy of metformin in the same system [23].

Consistent with our finding of SGLT2 protein in iPSC-derived cardiomyocytes, a recent study also identified SGLT2 in primary human cardiomyocytes from both diabetic and non-diabetic individuals [19]. The observed increase in SGLT2 expression in endothelial cells under diabetogenic conditions, but not in cardiomyocytes (Figure 1B,C), suggests a cell-specific response to diabetes, likely reflecting the distinct physiological roles and metabolic demands of these cell types under diabetogenic stress [41,42]. SGLT2 upregulation in endothelial cells has been primarily associated with hyperglycaemia [43]. Hyperglycaemia is sufficient to induce ROS production and the production of proinflammatory cytokines, including interleukin-6 and tumour necrosis factor-alpha, which activate redox-sensitive pathways to upregulate SGLT2 expression and fuel endothelial dysfunction [43].

A recognised limitation of human iPSC-derived cardiomyocytes is their relative immaturity compared to primary adult cardiomyocytes, raising the possibility that some findings in this study reflect features of the model rather than the true in vivo biology. Through immunofluorescent co-staining with SGLT2 and cell-specific antibodies, a predominant nuclear and perinuclear localisation with minimal cytoplasmic signal was observed across all three cell types. This pattern mirrors observations in primary human cardiomyocytes from both diabetic and non-diabetic individuals [19], indicating that the SGLT2 protein detected in iPSC-derived cardiomyocytes is unlikely to be a maturity-dependent artefact, and further supporting its expression in the human heart. To the best of our knowledge, the subcellular localisation of SGLT2 has not previously been characterised by immunofluorescence in isolated primary human endothelial cells or cardiac fibroblasts, and our findings represent a novel description of SGLT2 distribution in these cardiac non-myocyte populations.

Next, we examined the direct therapeutic effects of SGLT2 inhibitors on specific human cardiac cells. In 2D culture, iPSC-derived cardiomyocytes and endothelial cells exposed to diabetogenic conditions demonstrated decreased metabolic activity and a corresponding increase in cell death. Due to the limited regenerative capacity of cardiomyocytes, a reduction in cardiomyocyte number can markedly reduce ventricular contractile force [44]. Similarly, the loss of myocardial endothelial cells leads to microvascular dysfunction, increased vascular permeability and cardiac oedema [45,46]. Cardiac fibroblast activation also plays a vital role in diabetic cardiomyopathy pathogenesis; activation levels almost tripled under diabetogenic conditions, and cardiac fibrosis represents a critical early feature of the disease that progresses towards heart failure [47]. Additional proteomic analysis of cardiac fibroblasts or their extracellular matrix secretions may capture relevant changes in collagen deposition and other matrix constituents that further contribute to disease progression. Together, these 2D models recapitulated key cellular pathologies of diabetic cardiomyopathy.

To extend these findings to a more physiologically relevant context, we employed an established 3D multicellular cardiac microtissue model of diabetic cardiomyopathy [23,25,48]. Co-culturing iPSC-derived cardiomyocytes with non-myocytes creates a microenvironment rich in cardiac-specific signalling cues that enhances cardiomyocyte maturation and alignment, partially mitigating the inherent immaturity of iPSC-derived cardiomyocytes [49]. This platform allows precise control over cell type composition, with cardiomyocytes comprising 70% of the microtissue to support contractile function and structural stability, while endothelial cells and cardiac fibroblasts contribute to tissue compaction and contraction [50,51]. It has previously been validated across a range of cardiac disease contexts, including diabetes [23,25], drug-induced toxicity [34], ischaemia–reperfusion injury [32,33], and genetic cardiomyopathies [52]. Microtissues exhibited significantly reduced metabolic activity under diabetogenic conditions, consistent with the 2D findings. Notably, this reduction was less severe than that observed in 2D despite the longer treatment duration, suggesting potential protective effects from cell–cell interactions in the multicellular system [45]. Empagliflozin maintained microtissue metabolic activity at levels comparable to those of the control, representing a partial attenuation of the loss of metabolic activity under diabetogenic conditions.

Prolonged relaxation time under diabetogenic conditions is consistent with diastolic dysfunction, an early hallmark of diabetic cardiomyopathy that precedes systolic impairment [53]. The absence of a change in time-to-peak further supports the modelling of an early-stage phenotype. Contractile parameters did not differ between control and diabetogenic microtissues at 48 h, prior to any treatment intervention, indicating that the prolonged relaxation time and reduced beat rate variability observed at 96 h emerged between 48 and 96 h, consistent with the progressive nature of diabetic cardiomyopathy. Beat rate variability (RMSSD/RRint) was also significantly reduced under diabetogenic conditions, consistent with the reduced heart rate variability reported clinically in diabetic cardiomyopathy [54]. The large degree of variation in beat rate variability may reflect the presence of non-responders within the culture system, potentially arising from differences in the extent of metabolic adaptation to diabetogenic stress or batch-to-batch variability between individual microtissues. Together, these findings support the successful recapitulation of early diabetic cardiomyopathy phenotypes in the 3D model.

Empagliflozin failed to restore cardiomyocyte metabolic activity at any dose examined, suggesting that direct cardiomyocyte protection is not a primary mechanism under diabetogenic conditions. In contrast, empagliflozin rescued endothelial cell metabolic activity at all doses and reversed cardiac fibroblast activation, consistent with prior work demonstrating reduced profibrotic marker expression in empagliflozin-treated human cardiac fibroblasts [55]. Despite SGLT2 being detected and upregulated in endothelial cells under diabetogenic conditions, its absence from the plasma membrane suggests the involvement of alternative molecular targets. This is consistent with a growing body of evidence indicating that SGLT2 inhibitors act on the heart predominantly through off-target, SGLT2-independent pathways, including inhibition of the sodium–hydrogen exchanger 1 [14,15], SGLT1 inhibition [17], reduction in autophagy [16], inflammatory and neurohumoral modulation [18], as well as more recently described direct activation of pantothenate kinase 1 (PANK1) to promote coenzyme A synthesis and cardiac fuel metabolism [56].The findings of this study therefore support the currently proposed mechanisms by which SGLT2 inhibitors are offering cardioprotection via SGLT2-independent pathways [14,16,17,18].

**Figure 5:**
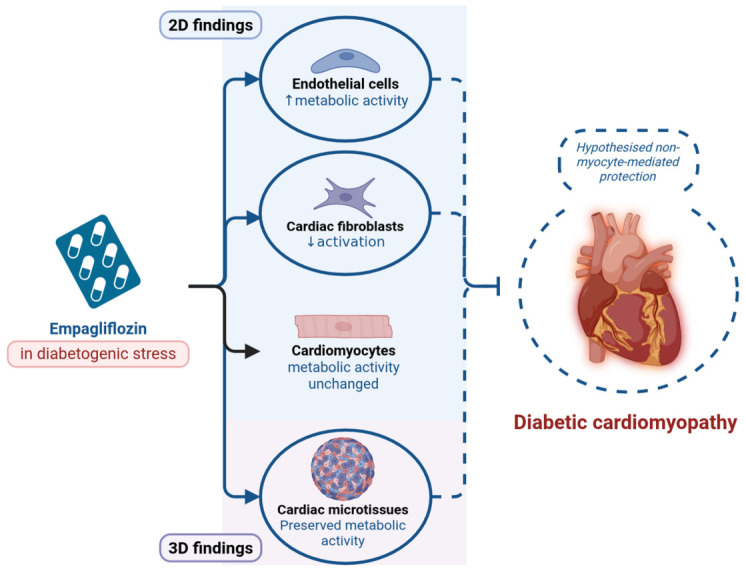
Proposed mechanism of action based on cell-type-specific responses to empagliflozin under diabetogenic conditions. Empagliflozin increased metabolic activity in endothelial cells and reduced the activation of αSMA-positive cardiac fibroblasts, while cardiomyocyte metabolic activity remained unchanged. Empagliflozin also preserved metabolic activity in cardiac microtissues, although this did not extend to the rescue of contractile dysfunction. As endothelial dysfunction and cardiac fibrosis are major contributors to diabetic cardiomyopathy, we propose empagliflozin-responsive non-myocytes as a potential route of cardioprotection in diabetic cardiomyopathy. Created in BioRender. Su, N. (2026) https://BioRender.com/2127anf accessed on 10 July 2026.

While empagliflozin maintained 3D microtissue metabolic activity at control levels, it did not rescue contractile dysfunction at the single dose examined. Cardiomyocytes represent the dominant cell type in the microtissue and showed no positive response to empagliflozin in 2D; therefore, the large cardiomyocyte population may have masked any therapeutic benefit occurring in the non-myocyte compartment. In addition, empagliflozin was introduced only after 48 h of established diabetogenic injury, representing a more demanding therapeutic scenario than a concurrent treatment paradigm, which may not have allowed sufficient time for non-myocyte-mediated benefits to propagate across the tissue. The increased complexity of 3D cell–cell interactions, including spatial organisation and paracrine signalling, may also have attenuated or redirected responses observed in isolated 2D cultures. Functional and therapeutic readouts are likely more susceptible to the effects of cellular immaturity than protein expression and localisation, and this may have contributed to the absence of a therapeutic rescue in cardiomyocytes and in the 3D cardiac microtissue. This partial response contrasts with the broader efficacy of metformin in preventing diastolic dysfunction in a related multicellular model that additionally incorporated autonomic neurons using a concurrent treatment paradigm [23]. Differences in model composition, treatment paradigm, and spatial cell–cell interactions between the two settings may account for this discrepancy. Figure 5 summarises these cell-type-specific responses to empagliflozin and the proposed non-myocyte-mediated route of cardioprotection.

This study has several limitations. Metabolic activity was used as the primary readout of therapeutic response and would benefit from complementary assessment of cell death by PI/Hoechst staining in the presence of empagliflozin. Similarly, the therapeutic effect of empagliflozin in cardiac fibroblasts was assessed at a single dose, and a full dose–response analysis in this cell type may reveal a more effective therapeutic concentration. Further mechanistic studies are also warranted to clarify the mode of action of SGLT2 inhibitors in these models, including SGLT2 knockdown, to confirm the extent to which these effects are SGLT2-dependent.

In summary, this study detected SGLT2 with nuclear and perinuclear localisation across iPSC-derived cardiac cell types, with no membrane-bound expression observed, contributing evidence to the ongoing debate regarding cardiac SGLT2 expression. Together, these findings support a model in which the clinically observed cardioprotection of SGLT2 inhibitors may be mediated, at least in part, through non-cardiomyocyte cell populations.

## Data Availability

The raw data supporting the conclusions of this article will be made available by the authors on request.

## Supplementary Materials

The following supporting information can be downloaded at https://www.mdpi.com/article/10.3390/jcdd13070341/s1, Video S1: Control; Video S2: Diabetes; Figure S1: Western blot of control and diabetogenic treated cardiomyocytes and endothelial cells; Figure S2: Effects of control and diabetogenic medium on 3D cardiac microtissues at 48 h.
