# Supplementary material for: Cardiac SGLT2 Expression and Cell-Type-Specific Responses to Empagliflozin in iPSC-Derived Models of Diabetic Cardiomyopathy
Source: J Cardiovasc Dev Dis. 2026 Jul 21;13(7):341. doi: 10.3390/jcdd13070341 (PMC13409743; doi:10.3390/jcdd13070341)
Supplement: Supplementary file 1 [file jcdd-13-00341-s001.zip › jcdd-4383081-supplementary.pdf]

Cardiac SGLT2 expression and cell-type-specific responses to empagliflozin in iPSC-derived models of diabetic cardiomyopathy

Nan Su[1, 2], Ren J Phang[1], Anne M Kong[1], Richard J MacIsaac[2, 3, 4], Shiang Y Lim[1, 5, 6, 7], Jarmon G Lees[1, 2, 6*]

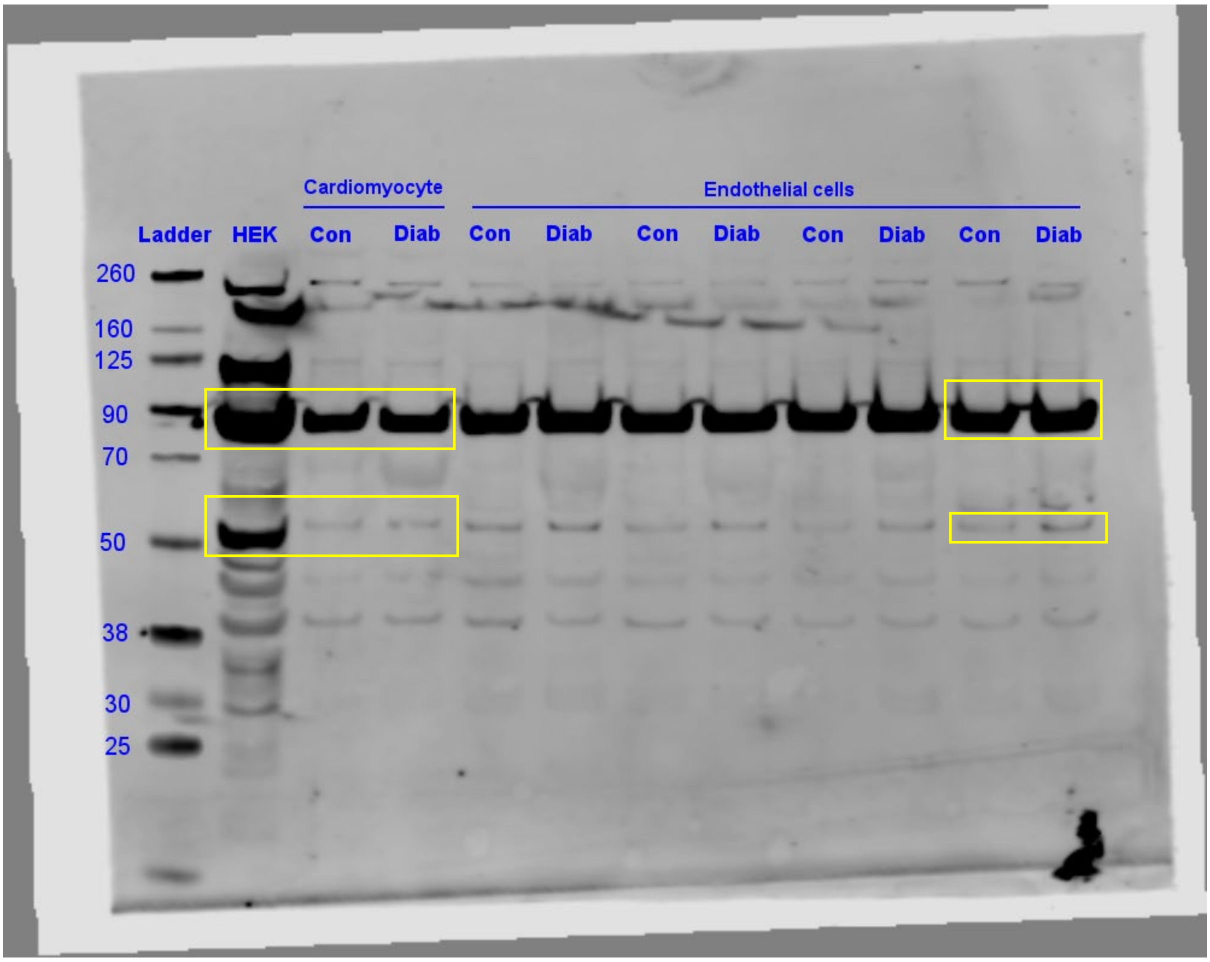

Supplementary Figure S1 Western blot of control and diabetogenic treated cardiomyocytes and endothelial cells. Yellow boxed regions are used as representative images in Figure 1A. Full western blot image of SGLT2 protein expression in cardiomyocytes and endothelial cells 48-hours after being cultured in either control (Con) or diabetogenic (Diab) conditions. Human embryonic kidney (HEK) cells were used as a positive control. Calnexin: ~90 kDa; SGLT2: ~46-75 kDa.

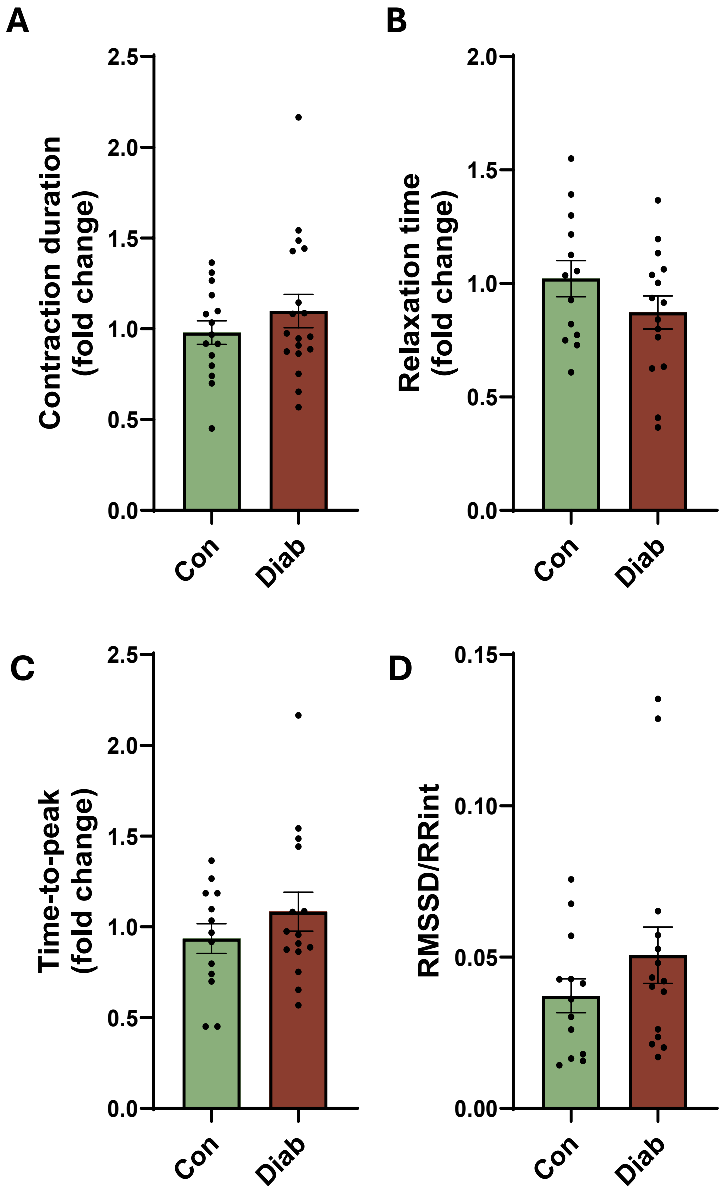

Supplementary Figure S2 Effects of control and diabetogenic medium on 3D cardiac microtissues at 48 hours. (A-D) Quantification of contraction duration (A), relaxation time (B), time-to-peak (C), and beat rate variability (RMSSD/RRint; D) in cardiac microtissues cultured under control (Con) or diabetogenic (Diab) conditions for 48 hours. Data are mean ± SEM; n = 13-15 from 5 independent experiments.
